# The Role of Open Space in Urban Neighbourhoods for Health-Related Lifestyle

**DOI:** 10.3390/ijerph110606547

**Published:** 2014-06-23

**Authors:** Katarina Ana Lestan, Ivan Eržen, Mojca Golobič

**Affiliations:** 1 Department of Landscape Architecture, Biotechnical Faculty, University of Ljubljana, Jamnikarjeva 101, Ljubljana 1000, Slovenia; E-Mail: Mojca.Golobic@bf.uni-lj.si; 2 National Institute of Public Health, Trubarjeva 2, Ljubljana 1000, Slovenia; E-Mail: ivan.erzen@gmail.com; 3 Medical Faculty, University of Ljubljana, Vrazov trg 2, Ljubljana 1104, Slovenia

**Keywords:** urban green areas, neighborhoods, health related behavior, urban planning, vulnerable user groups, quality of life

## Abstract

The research reported in this paper addresses the relationship between quality of open space and health related lifestyle in urban residential areas. The research was performed in the residential developments in Ljubljana, Slovenia, dating from the time of political and economic changes in the early nineties. Compared to the older neighborhoods, these are typically single-use residential areas, with small open spaces and poor landscape design. The research is concerned with the quality of life in these areas, especially from the perspective of the vulnerable users, like the elderly and children. Both depend on easily accessible green areas in close proximity to their homes. The hypothesis is that the poor open space quality affects their health-related behavior and their perceived health status. The research has three methodological phases: (1) a comparison between urban residential areas by criteria describing their physical characteristics; (2) behavior observation and mapping and (3) a resident opinion survey. The results confirm differences between open spaces of the selected residential areas as well as their relation with outdoor activities: a lack of outdoor programs correlates with poor variety of outdoor activities, limited to transition type, less time spent outdoors and lower satisfaction with their home environment. The survey also disclosed a strong influence of a set of socio-economic variables such as education and economic status on physical activity and self-perceived health status of people. The results therefore confirm the hypothesis especially for less affluent and educated; *i.e.*, vulnerable groups.

## 1. Introduction

It is increasingly recognized that place and space have an impact on human health and well-being and that health-related lifestyles of individuals are likely to be affected by their environment [1,2,3,4]. Along with an increase of the share of urban population the focus of the research in the field has shifted to the quality of urban environment [5]. According to World Health Organisation [6] the living conditions in the urban environment are the key to the health and well-being of its inhabitants. Evidence from the literature consistently indicates that there is an association between the built environment, health and well-being, and levels of physical activity [7,8,9].

The lack and poor quality of open/green space in urban neighborhoods can be a serious restriction for the wellbeing of the inhabitants as it does not support developing healthy life-styles, including spending time outdoors, walking, playing, *etc.* [1,8,10]. Spending time outdoors importantly reduces the exposure to indoor air, which is often polluted by the use of different artificial materials [11]. There is also a confirmed relation between spending time outdoors and a range of chronic diseases including obesity, diabetes type II, high blood pressure, coronary diseases, asthma, back and joint pains [12,13,14]. 

The research also shows a high response from the residents when offered easily accessible, high quality green open areas. In such circumstances, people tend to spend more time outdoors, which has a positive effect on life expectancy, health, happiness and general wellbeing, which is reflected in the efficiency of individuals and the whole society [15]. Accessibility to open and green space within their neighborhoods is especially important for certain groups of users such as the elderly, children, mothers with babies, and people with disabilities. The immediate availability of safe green space supports healthier physical and psychological development of children [16]. Evidence shows that children who have better access to such places are more likely to be physically active, and less likely to be overweight, compared to those living in neighborhoods with reduced access to such facilities. This has a long term effect since there is the direct correlation between using green open spaces in childhood and the state of health and lifestyle habits in adulthood [15]. Having direct contact with Nature tends to be a major challenge for children growing up in cities. [16]. Access to green space is also associated with greater longevity in older people [1]. Green areas of residential landscapes are essential for the vulnerable users, and cannot be expected to be replaced by green spaces found elsewhere in the city or in another period in life. Environment which is fit for the vulnerable groups is also more attractive for all users. The criterion of vulnerable groups is therefore increasingly applied in urban planning and design [15,17,18,19]. 

A review of recent practice regarding residential housing shows that appropriate open areas are largely missing from post-transition Slovenian collective residential developments [20]. A comparison with some older residential estates in Ljubljana shows that these were significantly better planned in terms of green open areas. The concept of a residential estate from 1960s was clearly structured, multifunctional and primarily socially oriented [21]. The “urban densification” paradigm, which has been prevailing in urban planning policies since the beginning of the millennium, in combination with the shortage of housing and its high prices resulted in collective housing environments with very limited and poorly equipped open and green space [17,18,19,22,23,24,19,22]. Together with vague standards and guidelines for open space, this resulted in low quality of new residential developments, lacking communal open spaces to accommodate the outdoor activities [18,20]. The findings from the national inquiry Housing in Slovenia from 2005 (Stanovanje v Sloveniji), show that lack of safe space for children’s playing and recreation are the second most important concern regarding living environment (after air pollution). These concerns are most exposed in the urban environments [25]. 

The aim of the research presented in this paper was to collect empirical evidence on the quality of open space in these newly built neighbourhoods and to reveal the linkages between the (lack of) provisions for outdoor activity and the life style adopted by the inhabitants—mostly youngest and oldest ones. It is assumed that poor quality and the lack of open green areas lead to poor forms of spatial uses and consequently to a less healthy lifestyle compared to areas with better quality of open areas. The behavioural style of residents is used as the indicator of health risks within the framework of public health [26]. An interdisciplinary approach was adopted, combining the field of urban planning with public health science, and building on the intrinsic link between health and planning from the 19th century, when planning primarily aimed at improvement of health conditions in the industrial cities [5,26]. Urban planning is one of the key health determinants, whereby neighborhoods are the basic building blocks within the city.

## 2. Methods

The research methodology adopted was divided in three phases according to the information gathering technique. The aim was to answer the following questions: What is the residential area urban design like? How many open green spaces are there in the area? Do open spaces in a residential area accommodate various land uses and activities according to the diverse needs of different age groups? (First methodological phase); in relation to the following question—what do people actually do in residential open spaces? (Second methodological phase); What is the residents’ opinion about their residential area?; How do they perceive it?; How do they feel there?; What is their life style?; How do they assess their well-being and health status? (Third methodological phase). Each methodological phase explores a certain layer of space: the first phase describes the physical qualities, the second phase tackles the behavioural-social aspects, and the third phase investigates its perceptive layer. 

At the beginning of the study, eight residential areas in Ljubljana were selected; four of which are more recent developments: Nova Grbina (1999), Viška Sončava (2007–2009), Celovški Dvori (2010) and Mesarska (2007), and the other four belong to the older generation of residential estates: VS4—Bonifacija (1972–1981), BS3 in Bežigrad (1976–1982), ŠS6 in Šiška (1967–1972) and VS1 in Trnovo (1972–1990). The research is limited to residential areas in Ljubljana in order to control for the macro-locational parameters, such as access to services and to green open areas in the city centre and its background. The first phase involved description and evaluation of open space design according to a set of urban development criteria.

**Figure 1:**
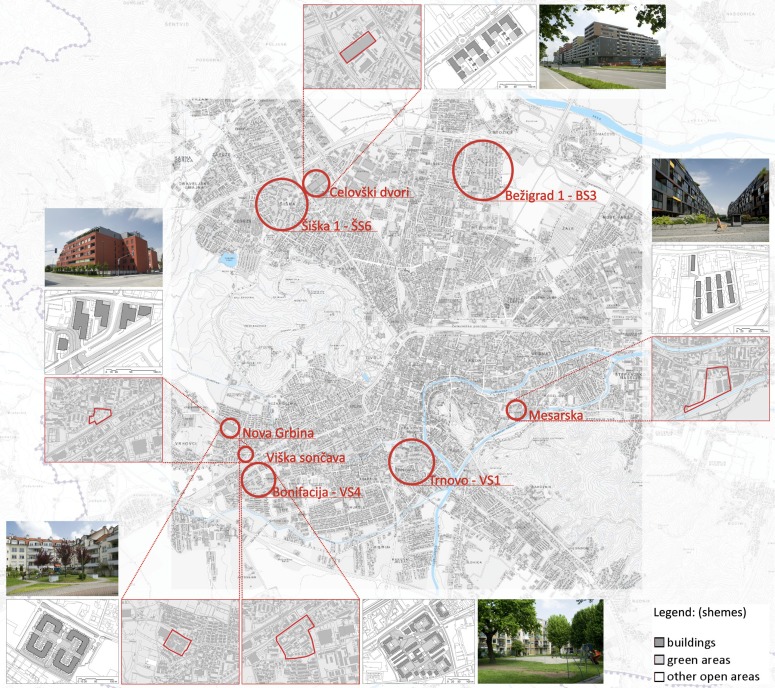
Locations of residential areas in Ljubljana, included in the first methodological phase of research. The residential areas included in the follow-up part of the study (Nova Grbina, Viška sončava, Bonifacija, Mesarska in Celovški dvori), are also illustrated in diagrams showing area outline, a schematic map with the map key, and a photo of the area.

Spatial, demographic and socio-economic aspects were considered as all three are closely related to lifestyles, which can be beneficial or harming to general health. The main indicators of urban environment quality on the neighbourhood level are related to residential density, variety of land use, accessibility of services, walking and cycling opportunities, urban green space, safe spaces for children play, for sitting and socializing [1,27,28]. The demographic criteria include the age structure of the residents, while the socio-economic criteria are the employment status and the level of education. Each criterion was allocated a set of indicators, evaluated individually for each residential area in reference to the prescribed or recommended values. These descriptions were used for the comparison between older and newer areas, and for selection of areas for further study. Since the focus of the research is new housing complexes we included all four into the further study. Among the older ones, Viška soseska 4—Bonifacija was assessed as the most appropriate example, as it is most comparable in size, population and type of buildings. It was therefore included in further study as a comparison (Figure 1). 

The second phase of the research involved the behavioural observations and mapping. This is one of the most commonly used methods in the research of urban open spaces [29,30]. Environmental psychology defines several types of behavioural maps; they can be behavioural matrices or maps in the true sense of the word. For the purpose of this research, both behavioural matrices and maps were used; the first ones serve as a quantitative description tool of individual activities, while the latter show the correlation between spatial structure and its uses. Before the beginning of fieldwork, a coding system for recording specific activities was defined based on a list of activities which could be predicted in advance. Unexpected or very specific types of activities could be added on the spot [29,30]. A schedule was set with three time periods of the day (from 10 am to 12 noon, from 1 pm to 3 pm, and from 5 pm to 7 pm). These time periods were chosen to include the highest variability of users: retired residents and mothers with children in the morning, children on their way home from school in early afternoon and all other user groups in late afternoon, e.g., working parents with children, older children, young couples, retired residents, and on the experience from previous research [29]. The residential areas were divided into observation units, which could be monitored within one sight of the observer. The number of such sub-areas varied between five and eight per residential area (Bonifacija eight, Viška Sončava five, Celovški Dvori six, Nova Grbina five and Mesarska six observed sub-areas). Every sub-area was then observed ten minutes within each time period. Ten days of observations were performed within three weeks in September 2012; including weekends. Ten observers were appointed to use the observation research method, one observer per residential area in each time period, covering all subareas in an individual residential area. Bonifacija residential area was covered by two observers per time period due to its size and the largest amount of sub-areas. A detailed schedule for ten days of observations was written to combine all residential areas, time periods and appointed observers. Each residential area was observed by all ten observers during the ten days of observations, in order to ensure objectivity of observation notes for all five areas. All shortlisted residential areas were observed at the same time, so the results show a comparative section of time and space. Apart one of the authors (Katarina Ana Lestan) of this paper, all researchers who did observations for this study were undergraduate students, final year students, and graduates from the Department of Landscape Architecture at Biotechnical Faculty, the University of Ljubljana. After the students had been selected, an introductory lecture was held in Biotechnical Faculty. Students were given the following information: aim of the study for which the observations were being made; explanation of the observations method and behavioural mapping, including an overview of what it helps us understand about specific spaces; and a presentation of two behavioural map types (matrix table, and spatial plan as a behavioural map in its true meaning). Further on, students were given instructions about who and what to observe, and when—the observations logistics (mid-week, weekend, two weeks altogether, three times per day on observation day). Students were also given information about equipment used for fieldwork (prepared maps of residential areas, matrices, paper, pens and a watch); they were acquainted with sub-areas, and the details about completing matrices and maps (noting the age-groups, duration of activity, symbols used to annotate activities on maps, *etc*). At the lecture, we assigned all students to specific observation times and residential areas, which were written down in the observations schedule; the students were also given the Goličnik and Ward Thompson article *Observations and behavioural mapping. A method for urban public open space research* [29]. The second stage included taking the participating students to Nova Grbina residential area, where a 10-min observation slot in a specific sub-area was demonstrated. At the third stage, all students were met together, and handed prepared folders with maps and matrices for three periods in the first day of observing, then we went separate ways to our allocated individual areas as allocated by the schedule. At the end of observations day we all met again to share experiences, questions, and challenges, and to hand in the completed sheets. The same activities were repeated on the second observations day. The following days students were met individually, and when handed their observation folders, emerging challenges were discussed, and that helped us stay in control of the quality of observations. We also kept in constant contact over phone and by email. Given the background of the students, they all understood the fieldwork very well; they were very good at linking the spatial plan of the area with the actual space, and drawing the observed activities on maps. 

The field observations were followed by data transfer into a Geographical Information System (GIS) and classification of the parameter categories. ArcView GIS 3.3 for Windows was used for this purpose. Every data input of an observed activity of one single person is described with parameters within the following categories: sex, activity, type of activity, category of activity, age, duration, time of day, part of the week, temperature, wind, air humidity, clear or overcast sky, and date. “Activity” is a descriptive category, which explains the action in more detail, while the “Type of Activity” assigns the detailed description to the nearest class, e.g., walking, playing, child care, spending time outdoors, *etc.* The “Category of Activity” groups all different activities observed during the field research into one of four general classes: “passive in space”, “active in transit”, “active in space”, “momentarily passive, otherwise in transit”. The category “Duration” labels only the activities in space, which are not transitory, and the “Time of Day” relates to one of the three daily time periods of observations. The remaining listed categories offer an insight into the current external conditions during the time of observations, which might affect the results of recorded activities and the overall use of the space. 

The third methodological phase involved survey of residents in all five areas. The survey was conducted within two weeks at the end of March and in the beginning of April 2013. The questionnaire was prepared in order to understand the subjective experience of residential environments from the perspective of residents and their health-related behavioural styles. Questionnaire structure is divided into two parts, and consists of mostly closed-ended questions. Questions regarding the life styles were selected from the questionnaire used in national survey CHMS, which was conducted in years 2001, 2004 and 2008 in Slovenia [12]. These include health self-evaluation, assessment of free-time physical activity, attitude to smoking and health self-evaluation of children. Questions regarding the residential area are drawn from RESTATE research (2007) [31], including inquiry about ownership status, property size, access to services, reasons for living in a certain residential area, satisfaction with the property and the area, perception of open spaces and the overall atmosphere in the area (contacts with neighbours, attachment to the area, *etc.*). The questionnaire additionally includes questions referring to the use of open space in their life style. Demographic data on gender, age, education and income levels, number of children and elderly people living in the same household, and employment status were also collected. People included in the survey were aged 18 and above. We aspired to achieve a balanced number of responses from each area (approximately 300). According to 2011 census, the total amounts of residents in individual residential areas are the following: Nova Grbina 543, Viška sončava 342, Celovški dvori 526, Mesarska 799 and Bonifacija 1613. Since the number of inhabitants differs it was not possible to provide the random selection of interviewees by selecting “every other door”, therefore an alternative measure was adopted. The sample included all inhabited apartments in the newer residential areas and every second in the older one. The interviewees were randomly selected by answering the question: “who in the household had had their birthday most recently”. The survey was conducted door-to-door: the interviewer personally handed the questionnaire to the interviewee, or left it in the mailbox if nobody was home, and collected it after it had been independently completed by the interviewee. In this manner, the sums of collected questionnaires per individual residential area are the following: Nova Grbina 74 (24.00% response rate), Viška sončava 86 (28.00%), Celovški dvori 132 (32.00%), Mesarska 107 (26.00%) and Bonifacija 155 (27.00%). The overall response rate was 28.00%. The known reasons for non-responsiveness include the lack of time, aversion to take part and bad mood, scepticism about benefitting from such a survey, and distrust towards interviewers, ringing their doorbells (some residents refused to open the door). 

SPSS predictive analytic software was used for the statistical data analysis. Correlations between certain key variables for the entire sample were calculated using Pearson’s coefficient. The focal questions of this article (“What do you normally do when you spend time outdoors within the area of your residential estate?”, “Time in hours per week spent outdoors in residential area”, “Time in hours per week spend on patios and balconies”, “What do you like the most about your residential area open space/what do you find disturbing?”) were interpreted by descriptive statistics. For the analysis of the means for questions “Recreational sport exercise in free time”, “Time in hours per week that children spend playing outdoors”, and “Self-assessed health of residents” ANOVA test was used in context of comparing means within the SPSS. The confidence interval (margin of error) has been calculated for the subsamples of 5 residential areas at 95% of the confidence level and 50% of the sample: Viška sončava; N = 87 (±8.30); Mesarska: N = 109 (±8.50); Celovški dvori: N = 130 (±6.80); Nova grbina: N = 70 (±10.60) and Bonifacija: N = 155 (±7.40) [32]. 

## 3. Results and Discussion

### 3.1. Description and Evaluation of Selected Residential Areas

The description of selected residential areas (Table 1) shows that the new residential complexes are much smaller in size even compared to Bonifacija, which is the smallest of the four older residential estates. The latter includes a local shop, community meeting facilities and a nursery with its own open area, as a part of the basic residential estate services. The plot ratio is higher in the new residential complexes than in Bonifacija, with Floor Space Index (FSI) exceeding the recommended values. Higher FSI means that a more residents will live in a given area [24], leading to a higher demand of open spaces within the residential area, most obviously noted in the case of Celovški Dvori. The above also implies that an appropriate amount of open spaces is not provided. 

FSI and the number of residents (Table 1) show the demand for using communal playgrounds, sports facilities (Table 2) and open green areas (Table 3). The surface of children playgrounds is inadequate in all studied cases; the situation is worst in Mesarska with 0.70 square metres of playground area per resident and a very high share—30.00% of children in the population [33].

**Table 1:** Evaluation of residential areas: spatial mesaure “residential area”, described by different indicators.

Measure	Indicator	Type of Measurement	Nova Grbina	Viška Sončava	Celovški Dvori	Mesarska	vs4 Bonifacija
			Value				
**Residential Area**	**surface area**	m^2^	30,700.10	20,443.54	24,364.42	30,299.44	83,744.53
	**number of residents**	number	543.00	342.00	526.00	799.00	1613.00
	**FSI—floor space index**	gross floor surface area/site surface area	1.14	1.17	2.79	1.80	0.68
	**site coverage**	gross ground floor surface area/site surface area	0.33	0.41	0.27	0.30	0.20

Comparison also shows a very low surface of playgrounds per resident in the case of Celovški Dvori, however they do provide communal sports facilities, unlike any other studied case. Bonifacija has the largest amount of playgrounds and sports facilities, which are also of the highest quality from the aspect of landscape design: large trees, bushes, a lawn, a hill. All new playgrounds lack such landscape features; in Viška Sončava playgrounds have no landscape elements at all. This is due to the practice of implementing green areas on the roofs of underground car parks, where the thickness of the soil layer does not support trees and designing park features [34]. One important consequence is poor or non-existent sun shelter.

**Table 2:** Evaluation of residential areas: spatial measure “equipment”, described by different indicators.

Measure	Indicator	Type of Measurement	Nova Grbina	Viška Sončava	Celovški Dvori	Mesarska	vs4 Bonifacija
			Value				
**Equipment**	**children’s playgrounds**	m^2^	1226.80	515.20	350.00	556.00	1867.00
		m^2^/ number of residents	2.30	1.51	0.67	0.70	1.16
	**sports facilities**	m^2^	0.00	0.00	393.00	0.00	658.00
		m^2^/ number of residents	0.00	0.00	0.75	0.00	0.41
	**car park areas**	number of parking places/apartment	1.80	1.50	2.00	2.00	0.90

For the aim of this research, the active-use areas were calculated (Table 3) excluding private areas, car parks and streets, and passive-use (private) areas. Active-use green areas are considered the ones substantially contributing to the overall quality of life and are equally accessible to all residents. The largest proportion of active-use open and green areas can be found in Bonifacija (29.48 m^2^ per resident). Mesarska (14,849.14 m²) has the highest amount of these areas among the neigbourhoods, followed by Viška Sončava and Celovški Dvori. However, even these numbers do not adequately describe the actual situation, since these areas are often the least experientially diverse; fully paved or tartan-covered surfaces, with no greenery and experiential value.

**Table 3:** Evaluation of residential areas: spatial mesaure “open and green areas”, described by different indicators (all open areas, functional areas—excluding private groundfloor atrium gardens, roads and external car park areas, passive areas—atriums, private use).

Measure	Indicator	Type of Measurement	Nova Grbina	Viška Sončava	Celovški Dvori	Mesarska	vs4 Bonifacija
			Value				
**Open and Green Areas**	**all open areas**	m^2^	20,669.51	12,115.06	14,284.42	20,959.14	66,946.51
	**functional areas (active-use areas)**	m^2^	8438.60	6829.84	12,976.17	14,849.14	47,557.51
		m^2^/number of residents	15.54	19.97	24.67	18.58	29.48
		m^2^/residential unit	26.70	27.54	15.58	21.58	52.61
	**passive areas**	m^2^	2426.09	1182.38	1308.25	6110.00	5190.00
		m^2^/number of residents	4.47	3.46	2.49	7.65	3.20

These results provide empirical evidence that the open space in post transition residential areas does not meet the standards either in terms of recommended value or in terms of comparison with older example. Bonifacija is a good practice example with its versatile urban furniture, green areas and adequate amount of open areas, offering various spatial uses and activities for all age groups. There are also differences among the new neighbourhoods: open space quality and quantity is best in Nova Grbina. Although open spaces with many children’s playgrounds and a sports field in Celovški Dvori offer a wide variety of uses, the high density of the area, and poor greenery restrict the possibilities for open space use. The open spaces of Viška Sončava and Mesarska, which lack any green areas, are the poorest examples within the selected residential areas.

### 3.2. Behavioural Observations and Mapping

The graphical presentation of observations enables an insight of spatial use according to “category of activity” and “age group”. The activities included in the category “active in space”, are e.g., child’s play, playful running and trampoline jumping; and the activities defined as “passive in space” include e.g., minding children while they play, sitting on a bench, chatting, *etc.*

**Figure 2:**
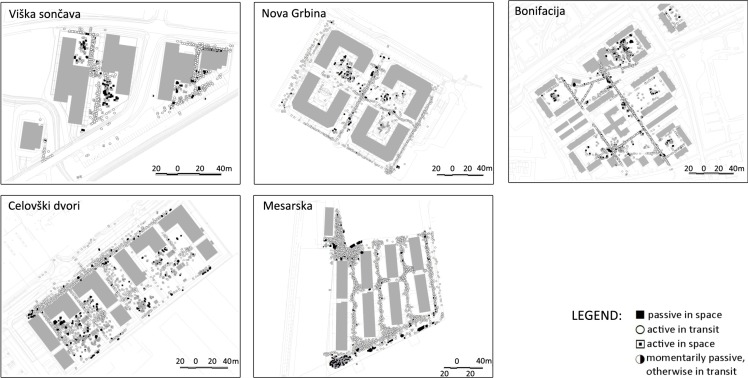
Graphical presentation of observation results according to the “Activity” category.

**Figure 3:**
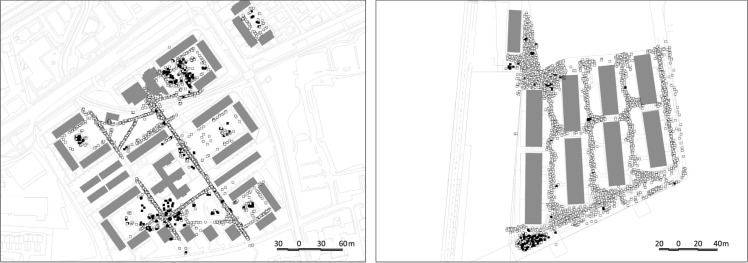
Left: Results of observations and behavioural mapping in Bonifacija showing the category “active in space”, filtered to show age groups 1 and 2 (up to 12 years) (black spots) in relation to other activities observed in the same time (white spots). Right: Results of observations and behavioural mapping in Mesarska showing the category of activity “active in space”, filtered to show age groups 1 and 2 (up to 12 years) in relation to other activities observed in the same time.

In general, the observation method revealed that all open spaces in the newer residential areas are mostly used by the youngest users, while the lack of facilities for other age groups (e.g., playgrounds for older children, sports fields, and shaded and safe spaces for retired residents) results in less (active) use among these users. Despite the poor spatial street furniture and landscape features of the newer residential areas, there were many open space users observed in these areas. However, the majority of observed users were engaged in transitory activities, indicating that open spaces do not attract or not even enable activities of spending more time in the area. Based on the observations, Bonifacija again provided an example of good practice: transitory uses were limited to the footpaths, while a variety of activities were observed in different areas offering possibilities (benches, playgrounds, trampolines, diverse landscape features), a higher number of retired residents were observed who spend their time outdoors, and a wider variety of children’s play (Figure 2 and Figure 3).

The graphic presentation of the results does not entirely disclose all the variety of children play in Bonifacija. The active children are equally distributed across the open area; with a large share of children’s play activities observed outside of playgrounds, especially in the areas with diverse landscape features such as the edges between open spaces and vegetation and in combination with undulating terrain. As also stated in Kučan *et al.* [35], imaginative child’s play is always stronger in places, which do not strongly define the uses. On the other end of the spectrum, the residential landscapes of Mesarska are (similar to other new areas) predominantly used for transit, while children are confined to one specific area, the children’s playground, which is used beyond its capacities and will increasingly continue to be overused when new residents occupy the empty flats. The comparison between Bonifacija and Mesarska confirms the hypothesis that poor programme equipment and the lack of open areas leads to a reduced variety of spatial uses.

### 3.3. Questionnaire

The interpretation of the results obtained by the third part of research addresses the question of: “whether and how the design of open spaces affects the residents’ behavioral patterns?”. While open space design cannot directly affect the health related behavioral patterns of eating, smoking, alcohol consumption, drugs intake, *etc.*, they may however affect the time spent outdoors, and the physical activity of children and the elderly, who depend on the proximity of green spaces to their homes. Therefore data interpretation focuses on the questions, addressing the amount of free time spent for recreation, type of outdoor activities and the perception of residential area, health status and general well-being. Due to the fact that open space is not the only behavioral pattern indicator which supports or harms public health, it was also necessary to reflect on correlations with other research variables, which might be significant for this matter. 

On average, the strongest correlations proved to be the ones between the satisfaction with the apartment and the satisfaction with the residential area; between the apartment/residential area satisfaction and the self-assessed health of residents and their children; between the economic status/the level of completed education and the self-assessed health; between the economic status and time spent in very intensive sport exercise; and between self-assessed health and very intensive sport exercise (Table 4).

The physical activity of the inhabitants was assessed by the question “Recreational sport exercise in free time”, which includes intensity (very intensive sport exercise, moderate exercise and walking) and duration (expressed in minutes per day), and the frequency (number of days per week).

This question is not directly related to the quality of residential area open space, as people normally take part in these activities elsewhere, outside of the residential area (fitness classes, dancing, fast cycling, running, *etc.*). The layout of Bonifacija (Figure 1) does show more opportunities for recreation on site compared to other areas, however results show the lowest number of days per week spent engaged in recreational physical activity, compared to other residential areas. This is not surprising taking into account different age structure of Bonifacija population (all age groups, including many elderly residents) compared to the newer residential areas (mostly young families) [33]. The structure of the population (age, education, income) is a more important determinant of physical activity than neighborhood characteristics [12]. In the case of Bonifacija, a correlation can be only found between variables representing very intensive physical activity and age (*p* = 0.01), with the amount of activity reducing with increasing age. The highest amount of days per week spent in very intensive and moderate physical activity is, on average, claimed by the residents of Viška Sončava and Mesarska. Both cases show a statistically significant correlation between physical activity and the highest completed level of education (Viška Sončava: very intensive sport exercise *p* = 0.00, moderate exercise *p* = 0.04, walking *p* = 0.03; and Mesarska: very intensive sport exercise *p* = 0.01, moderate exercise *p* = 0.00, and walking *p* = 0.04) (Table 5). There was no significant correlation found between physical activity and education or income level within the respondents from Celovški Dvori and Nova Grbina, however this correlation was confirmed in the whole sample (Table 4).

**Table 4:** Correlations between certain key variables for the entire sample.

		Satisfaction with the Apartment	Satisfaction with the Residential Area	Economic Status	Very Intensive Sport Exercise	Level of Completed Education	Self-Assessed Health of Residents	Self-Assessed Health of Residents Children
**Satisfaction with the Apartment**	**Pearson Correlation**	1.00	0.61 (**)	-0.01	0.02	0.02	0.18 (**)	0.21 (**)
	**Sig. (2-tailed)**		0.00	0.75	0.58	0.55	0.00	0.00
	**N**	542.00	542.00	527.00	542.00	541.00	536.00	228.00
**Satisfaction with the Residential Area**	**Pearson Correlation**	0.61 (**)	1.00	0.05	0.01	0.08	0.22 (**)	0.23 (**)
	**Sig. (2-tailed)**	0.00		0.27	0.82	0.06	0.00	0.00
	**N**	542.00	543.00	528.00	543.00	542.00	537.00	229.00
**Economic Status **	**Pearson Correlation**	-0.01	0.05	1.00	0.16 (**)	0.52 (**)	0.25 (**)	0.01
	**Sig. (2-tailed)**	0.75	0.27		0.00	0.00	0.00	0.90
	**N**	527.00	528.00	535.00	535.00	534.00	529.00	230.00
**Very Intensive Sport Exercise**	**Pearson Correlation**	0.02	0.01	0.16 (**)	1.00	0.21 (**)	0.27 (**)	0.03
	**Sig. (2-tailed)**	0.58	0.82	0.00		0.00	0.00	0.68
	**N**	542.00	543.00	535.00	550.00	549.00	544.00	236.00
**Level of Completed Education **	**Pearson Correlation**	0.02	0.08	0.52 (**)	0.21 (**)	1.00	0.33 (**)	0.06
	**Sig. (2-tailed)**	0.55	0.06	0.00	0.00		0.00	0.36
	**N**	541.00	542.00	534.00	549.00	549.00	543.00	236.00
**Self-Assessed Health of Residents **	**Pearson Correlation**	0.18 (**)	0.22 (**)	0.25 (**)	0.27 (**)	0.33 (**)	1.00	0.24 (**)
	**Sig. (2-tailed)**	0.00	0.00	0.00	0.00	0.00		0.00
	**N**	536.00	537.00	529.00	544.00	543.00	544.00	234.00
**Self-Assessed Health of Residents Children**	**Pearson Correlation**	0.20 (**)	0.23 (**)	0.01	0.03	0.06	0.24 (**)	1.000
	**Sig. (2-tailed)**	0.00	0.00	0.90	0.68	0.36	0.00	
	**N**	228.00	229.00	230.00	236.00	236.00	234.00	236.00

Notes: ** Correlation is significant at the 0.01 level (2-tailed).

According to the guidelines of the WHO [36], the minimum amount of physical activity required for a healthy lifestyle, is 75 min of intensive sport activity per week. The inhabitants of Mesarska come close to meeting these conditions, with almost 60 minutes of this kind of activity on average per week. In Viška sončava interviewees spend only 45 minutes engaged in such exercise per week. The residents of Celovški dvori, Nova grbina and Bonifacija exercise even less. While only recreational exercise was included in the survey, these guidelines can also be partially met by the physical activity at work, running domestic errands and physical movement involved on the way to work or a study place. 

**Figure 4:**
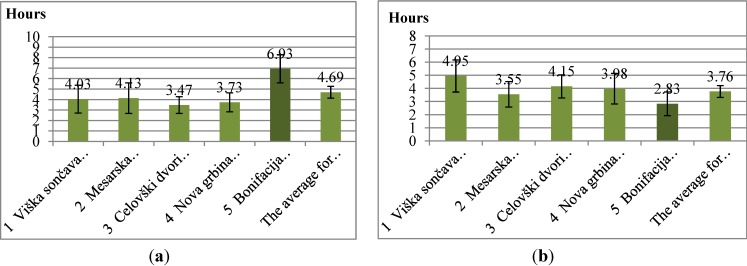
Left: Time in hours per week spent outdoor in residential area. Right: Time in hours in spend in patios and balconies (95% Confidence Interval of the Difference).

Notes:  newer residential developments;  older residential estate as a comparison with better quality of open areas.

**Figure 5:**
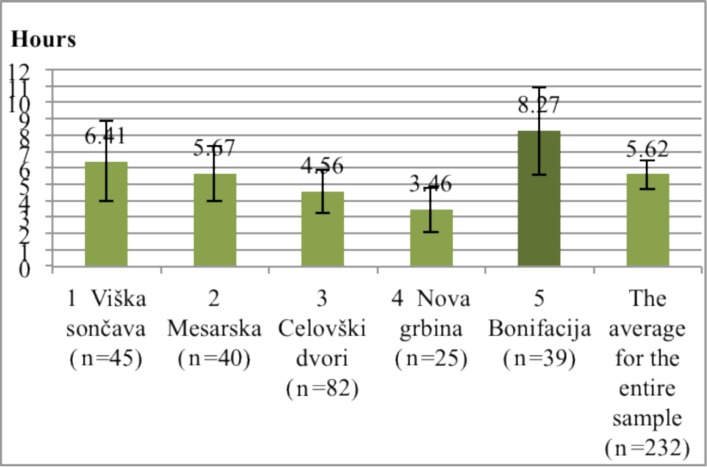
Time in hours per week that children spend playing outdoors (95% Confidence Interval of the Difference).

Notes:  newer residential developments;  older residential estate as a comparison with better quality of open areas.

In contrast to the residents of newer developments which claim to spend most time on patios and balconies, the residents of Bonifacija report to spend the most time in the outdoor spaces (Figure 4). The amount of time that children spend playing outdoors (Figure 5) is also the highest in Bonifacija (8.27 h per week). These results coincide with the description and evaluation of open space and the observations of behavior. The least amount of such time is reported in Nova Grbina (3.46 h per week). While Nova Grbina boasts with the best open space among new residential areas, it also has inhabitants with the highest education level (64.30% completed the highest level of education). This can explain why the children spend the least hours playing outside. Children of highly educated parents tend to take part in more after-school activities, leaving them with less time available for play [37].

**Table 5:** Recreational sport exercise in free time for very intensive sport exercise (1), moderate exercise (2) and walking (3), showing average amount of days per week (0–7 days).

Sample		Viška Sončava			Mesarska			Celovški Dvori			Nova Grbina			Bonifacja		
intensity		1	2	3	1	2	3	1	2	3	1	2	3	1	2	3
**age**	**<39**	1.79	2.26	3.17	2.26	2.12	3.36	1.19	2.11	3.77	2.06	1.78	3.34	1.55	1.76	2.80
	**40–54**	1.09	1.85	3.41	1.68	2.57	1.88	1.01	1.28	2.90	1.19	1.79	1.90	0.78	1.12	2.68
	**<55**	0.59	2.46	3.55	0.48	1.24	1.30	0.11	0.57	5.48	0.26	1.50	3.89	0.60	1.24	3.65
**Total**		1.44	2.20	3.29	1.89	2.05	2.80	1.05	1.73	3.64	1.25	1.71	2.94	0.97	1.41	3.20
**Sig.**		0.03	0.72	0.82	0.00	0.16	0.01	0.21	0.06	0.04	0.00	0.84	0.01	0.01	0.32	0.17
**level of education**	**primary education**	0.00	1.80	3.20	3.00	4.50	3.50	0.71	1.43	4.09	0.00	0.00	0.00	0.48	1.21	4.34
	**vocational degree **	1.33	4.00	5.17	0.00	0.00	0.00	0.94	1.72	3.65	0.51	1.49	1.98	0.65	0.94	2.91
	**secondary level**	1.96	2.13	2.96	2.52	1.86	3.61	1.07	1.49	3.01	1.30	1.50	2.89	0.93	1.36	2.77
	**higher education**	1.76	1.90	2.94	1.69	2.16	2.72	1.89	2.95	3.97	1.23	1.75	2.99	1.59	1.87	2.92
**Total**		1.44	2.19	3.26	1.87	2.10	2.84	1.05	1.73	3.64	1.21	1.66	2.90	0.97	1.39	3.16
**Sig.**		0.00	0.04	0.03	0.01	0.00	0.04	0.14	0.12	0.40	0.61	0.86	0.72	0.02	0.31	0.08
**total household monthly income**	**<550 €**	1.15	2.21	4.04	2.92	3.63	3.99	0.90	1.00	2.10	1.19	0.94	1.52	0.74	0.81	2.42
	**551 €–1.300 €**	1.04	2.02	3.10	1.32	1.49	2.21	0.87	1.98	4.46	0.53	1.55	2.80	0.92	1.68	4.17
	**1.301 €–2.200 €**	1.65	2.39	3.96	2.02	2.18	2.99	1.66	1.87	2.88	1.57	2.00	3.44	1.07	1.42	2.04
	**<2.201 €**	2.04	1.89	2.44	1.68	2.03	2.55	2.50	3.50	3.50	1.39	1.46	2.59	1.53	1.22	2.32
**Total**		1.43	2.08	3.22	1.85	2.12	2.78	1.04	1.75	3.61	1.21	1.66	2.90	0.95	1.39	3.21
**Sig.**		0.16	0.91	0.10	0.18	0.11	0.41	0.22	0.25	0.00	0.10	0.54	0.29	0.62	0.25	0.00

The responses of residents with highest completed education show us that the average time of children’s play in outdoor residential area is the lowest in the case of Nova Grbina (Table 6). The average time spent in outdoor play activities is 5.19 h per week for the entire sample; therefore children living in Nova Grbina play substantially less than the average child from other areas.

**Table 6:** Time in hours per week that children spend playing outdoors according to the higher education of parents.

**Higher Education**	**Viška Sončava**	**Mesarska**	**Celovški Dvori**	**Nova Grbina**	**Bonifacija**	**Total**
	6.65	5.60	3.33	3.28	6.97	5.19
**Sig.**	0.02	0.87	0.22	0.80	0.87	0.07

Correlation between the highest completed education level of parents and the time their children spend playing outdoors in an individual residential area is in fact only statistically significant in the case of Viška Sončava (*p* = 0.02), while in the case of other residential areas there has been no statistical significance found. This could be a consequence of a small statistical sample. A weak point of this study is small sample size. Confidence intervals have a larger scope on Figure 5, because only people with children had answered that question. Error is less likely at Figure 4, where the entire sample of respondents is represented (see supplementary file “Confidence intervals”).

**Figure 6:**
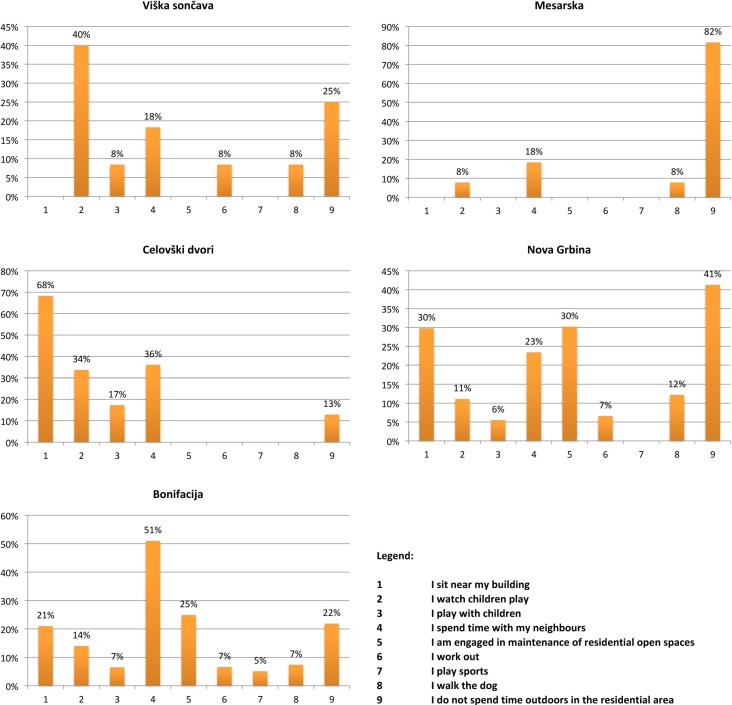
Answers to the question: “What do you normally do when you spend time in open spaces of your residential area?” by retired residents.

Besides children, the elderly were another focus of the research. A separate analysis was performed for the retired residents asked: ‘What do you normally do when you spend time outdoors within your home area?’ The majority (51.00%) of 57 retired residents of Bonifacija socialise with their neighbours; a quarter (25.00%) are engaged in maintenance of residential open spaces or sit near their building (21.00%); 14.00% of respondents accompany children at play; followed by exercise (7.00%), engage in a sports activity (5.00%), play with their children (7.00%) or walk their dog (7.00%). 22.00% of retired residents claim not to spend time outdoors within the residential area. All of the activities offered by the multiple-choice answers have been represented in the replies of Bonifacija residents. However, with the exception of Nova Grbina, the results gathered from the newer residential areas show that many activities from the list are not represented in the answers (Figure 6). 

In general, it can be concluded that retired residents do not spend time outdoors in newer residential areas, and if they do it is for accompanying children. The interpretation of activities that retired residents engage in must take into consideration the fact that they represented a very small proportion of interviewees (12.00% in Viška sončava, 8.40% in Mesarska, 7.20% in Celovški dvori, 22.90% in Nova grbina, and 39.30% in Bonifacija), therefore these samples are not statistically representative of the population. 

Another question related to perception of their home residential area referred to the aspects of open spaces they like best/ find disturbing? (Table 7 and Table 8) Highlighted are the answers, which were selected most frequently by residents of all five areas. The well-designed green areas were most frequently chosen in Nova Grbina (61.40%) and Bonifacija (71.30%). 

**Table 7:** What do you like the most about your residential area open space? (multiple choice answers).

	Well Maintained Green Areas	Good Quality Footpaths	Benches or Tables, Where We can Spend Time Together	Many Trees	Well Maintained Children’s Playgrounds	Good Quality Sports Fields	Peace	Sense of Security	Easy Car Parking	Count
**Viška Sončava**	17.50%	20.70%	6.50%	6.50%	35.80%	9.50%	28.60%	36.60%	80.20%	87.00
**Mesarska**	22.70%	32.80%	4.20%	8.90%	21.20%	1.40%	71.80%	27.10%	45.20%	106.00
**Celovški Dvori**	23.60%	31.80%	21.10%	3.60%	48.30%	18.30%	10.00%	5.20%	66.00%	120.00
**Nova Grbina**	61.40%	22.70%	9.30%	11.40%	28.50%	9.90%	61.30%	63.40%	63.20%	70.00
**Bonifacija**	71.30%	34.00%	23.40%	48.10%	47.00%	9.70%	56.70%	31.40%	21.50%	154.00
**Total**	41.10%	29.60%	14.50%	18.90%	38.00%	10.00%	45.30%	29.70%	51.10%	537.00

**Table 8:** What disturbs you the most about the open spaces in your residential area? (multiple choice answers).

	Unkept Green Areas	Untidy Footpaths	Not Enough Benches or Tables for Spending Time Together	Not Enough Trees	Poorly Maintained Children’s Playground	Poor Quality Sports Fields	Inadequate Safety	Difficult Car Parking	Nothing	Noise from the Playgrounds	Traffic Noise	Count
**Viška sončava**	10.70%	3.00%	11.80%	34.30%	8.80%	4.90%	20.90%	8.70%	15.80%	15.80%	58.80%	86.00
**Mesarska**	25.00%	13.10%	27.10%	58.60%	11.90%	10.70%	11.00%	38.60%	6.10%	2.60%	5.40%	106.00
**Celovški dvori**	26.30%	4.00%	15.20%	51.40%	8.40%	2.80%	59.50%	8.40%	8.10%	36.90%	26.70%	127.00
**Nova grbina**	2.50%	6.60%	14.50%	20.70%	1.20%	5.00%	3.10%	6.90%	47.60%	5.10%	13.50%	69.00
**Bonifacija**	7.00%	8.10%	24.90%	10.00%	6.90%	6.60%	10.30%	37.60%	28.80%	3.20%	27.60%	149.00
**Total**	15.20%	7.10%	19.60%	34.70%	7.80%	6.00%	22.90%	22.30%	19.70%	13.30%	26.20%	536.00

Poorly maintained green areas in Celovski Dvori (26.30%) and Mesarska (25.00%) (in line with the inventory data of the areas) are rated as disturbing, while in Bonifacija only 7.00% of respondents rated unkept green areas as disturbing (Table 8). Other responses also confirm the inventory findings: the residents of Mesarska express a wish for more benches and tables for socialising (27.10% of respondents find this lack disturbing), presence of many trees is appreciated by the residents of Bonifacija (48.10%), and the residents of newer residential areas complain about not having enough trees in their area (34.30% of respondents in Viška Sončava, 58.60% in Mesarska, 51.40% in Celovški Dvori, and 20.70% of respondents in Nova Grbina). Children’s playgrounds are perceived to be of lowest quality in Mesarska, (12.00% find poorly maintained children’s playgrounds disturbing), another finding corresponding to least square meters of playground areas per resident. Outdoor sports facilities are also assessed as least attractive in the case of Mesarska (10.70%) (there is an improvised football field, showing the need for such areas). Celovski Dvori clearly stand out in terms of security problems (social housing, vandalism, drugs, *etc.*), 59.50% residents selected the answer “inadequate safety” as disturbing, the noise from the playgrounds (echo effect in between blocks of flats), and a general lack of peacefulness (noise from the playgrounds and traffic noise together represent 63.60% respondents’ answers). The best area in terms of safety and lack of disturbances is Nova Grbina (47.60% are not disturbed by anything, the answer “peace” was selected by 61.30%, and “a sense of security” was selected by 63.40% of respondents). Ease of parking is valued by residents in newer developments (The ease of car parking is valued by the residents of Viška Sončava 80.20%, Mesarska 45.20%, Celovški Dvori 66.00%, and Nova Grbina 63.20%), while in the older area of Bonifacija, finding parking spots was assessed as problematic (the highest percentage of answers regarding the difficulty of car parking, 37.60%).

Self-evaluation of health is an established tool for assessing the general state of health indicating low capability, functional competence, frequency of illness, and mortality of population. Many factors influence the health self-evaluation, including age and the socio-economic status. [12]. The actions for improving the self-evaluation of health and thus to reduce social inequality are needed [38,39].

The respondents assessed their health status on Likert scale: “Very poor”, “Poor”, “Average”, “Good”, and “Very good”. The overall sample shows the expected statistically significant correlation between the health self-evaluation and age (*p* < 0.05). Apart from age, level of education is a significant variable in the case of health self-evaluation (*p* < 0.05). The whole statistical sample also showed a correlation with economic status (*p* < 0.05) (Table 9). Health self-evaluation in the case of the whole sample also indicated a correlation with physical activity: a higher self-evaluation of health was shown by respondents who spend more time engaged in very intensive physical activity (*p* < 0.05) (Table 4). 

Health self-evaluation and the residential area are not directly correlated. Most residents assessed their health as “Very good” in Viška Sončava (32.20%) and Mesarska (32.00%) residential areas, which are the worst in terms of provisions for outdoor activities. In Bonifacija as the best example, (Figure 1), only 14.20% of the residents self-assessed their health to be “Very good”, which is the lowest percentage compared to other residential areas. Results from Viška Sončava and Mesarska show a correlation between health self-evaluation and education (Viška Sončava *p* = 0.00; Mesarska *p* < 0.05). In both residential areas there is the highest amount of residents with a completed highest level of education, compared to other residential areas as well as within the area (Viška Sončava 47.70% and Mesarska 64.70%). Average health self-evaluation in the group of higher education in Viška sončava is 4.20 (±0.26) and in Mesarska residential area 4.27 (±0.17). There is also a correlation between health self-evaluation and education in the case of Bonifacija (*p* = 0.01), however in this case the lower self-assessment can be explained by the age of residents (*p* < 0.05). This area accommodates the highest amount of elderly residents (34.80% of residents aged 61 years or more). The lowest self-assessment of health was shown by the residents of Celovški Dvori (“Very poor” 10.80% and “Poor” 8.10%). The latter correlated to the completed education level of its residents (*p* = 0.01). Many units in Celovški Dvori are social housing, with highest amount of residents with completed elementary level of education (32.30%) compared to other residential areas. The residents of Nova Grbina showed the highest amount of responses self-evaluating their health as “Good”. While this residential area offers many spatial options for spending time outdoors and pursuing a health-supporting lifestyle, it is more likely that such a high level of self-evaluated health is related to the high economic standard of the residents (*p* = 0.01) (Table 9). 

**Table 9:** Self-evaluation of health according to variables of age, level of completed education and total household monthly income (* 95% Confidence Interval of the Difference).

	Sample	Viška Sončava	Mesarska	Celovški Dvori	Nova Grbina	Bonifacja	Whole Sample
**Age**	**<39**	4.18	4.22	3.60	4.06	4.13	4.01
	**40–54**	3.61	4.10	3.58	4.20	3.71	3.81
	**<55**	2.96	3.57	2.29	3.43	3.33	3.26
**Total**		3.84	4.10	3.49	3.94	3.68	3.77
**Sig.**		0.00	0.01	0.00	0.00	0.00	0.00
**total Household monthly income**	**<550 €**	4.11	4.11	3.18	3.67	3.59	3.58
	**551 €–1.300 €**	3.40	3.68	3.55	3.68	3.59	3.56
	**1.301 €–2.200 €**	4.27	4.15	3.61	3.96	3.72	3.95
	**<2.201€**	4.19	4.26	4.00	4.31	4.16	4.24
**Total**		3.87	4.10	3.48	3.95	3.65	3.77
**Sig.**		0.01	0.07	0.45	0.01	0.23	0.00
**Level of education**	**primary education**	3.00	4.50	3.40	/	3.34	3.39
	**vocational degree**	3.67	3.00	2.94	3.51	3.53	3.29
	**secondary level**	3.91	3.81	3.71	3.75	3.78	3.78
	**higher education**	4.20	4.27	3.97	4.07	3.97	4.13
**Total**		3.86	4.10	3.49	3.95	3.69	3.78
* Lower/Upper		3.63/4.09	3.94/4.25	3.28/3.69	3.80/4.09	3.54/3.81	3.70/3.86
**Sig.**		0.00	0.00	0.01	0.05	0.01	0.00

Exploring responses of the age group 55 years and above, the residents of Bonifacija do not report the lowest average health self-evaluation level (3.33) compared to the other four residential areas. Lower health self-evaluation values than the ones in Bonifacija are shown by the residents in Viška Sončava (2.96) and Celovški Dvori (2.29). The highest levels of health self-evaluation by the residents aged 55 and above are reported by residents in Mesarska (3.57) and Nova Grbina (3.43). Within all five individual residential areas there is a statistical correlation between age and health self-evaluation. Interpreting results in the age group of 55 years and higher, it has to be taken into consideration, that the number of respondents was substantially lower in newer residential areas than in Bonifacija. There were only 10 to 18 respondents over 55 years of age in newer areas, while there were 74 respondents belonging to that age group in Bonifacija. Average health self-evaluation of residents above 55 in Bonifacija is above the average of the total statistical sample (3.26) (Table 9). The level of self-evaluation may partially be this high due to a good spatial design of the Bonifacija area, where there were many elderly people observed spending time outdoors during the second methodological stage.

In terms of Children’s health, no significant difference was observed, generally being assessed as “Good” or “Very good” in all five residential areas.

## 4. Conclusions

In order to comprehensively interpret the role of residential landscapes for life styles of their users, it is essential to connect the physical, social and symbolic spatial variables, which reveal the invisible links between the structure of space, behaviour and perception [40,41]. By highlighting the determinants, which impact occupants’ health related life styles, the results of this research contribute knowledge to the hypothesis that poor quality and the lack of open green areas lead to poor forms of spatial uses and consequently to a less healthy lifestyle. 

As regards *spatial determinants of health* (phase 1), the results confirm and provide empirical support for the statements regarding the characteristics of post transition residential areas:

- Excessive density;
- Lack of open/green areas;
- Poor design and furniture of outdoor space.

These residential areas are well equipped only for the youngest children, while needs of older children and especially elderly residents are neglected. 

The main findings regarding the *behavioural determinants of health* (phase 2) provide evidence of linkage between the quality of open space and the variety and length of its use:

- This is especially significant for children, which tend to actively use different spaces across the area, if they are suitable (vegetation, open meadows and undulations in topography) and do not restrict their activity on playgrounds with pre-defined ways of use.
- On the other hand, in residential areas with inadequately equipped and poorly landscaped open space, the observations show more transitory uses.
- Retired residents in new areas nearly exclusively spend time outdoors accompanying children, while in Bonifacija, they engage in more variety of activities. This indicates that the open spaces in newer residential areas do not meet the needs of the elderly.

*Perceptual determinants of health* (phase 3) are indirect, and affect the behavioural patterns of occupants:

- Residents in new developments perceive the lack of trees and estimate the green open areas as less attractive compared to those in older one.
- Residents in new developments prefer to spend time in private atriums and balconies.
- The highest amount of time spent in play activities is claimed by the parents of children from Bonifacija, which also has the highest amount and quality of outdoor space.
- Behavioural styles, especially engagement in physical activity, health self-evaluation and satisfaction with living environment, are strongly correlated with economic status and education.

Although the research confirms linkages between spatial, behavioural and perceptive determinants of life style, it is additionally influenced by many other variables. The claim that better use of open space also leads to a generally healthier lifestyle and better self-assessed health could therefore not be entirely confirmed. The influence of other determinants such as economic status, age and education is too strong to allow for any conclusive statements.

The hypothesis assumes that health related life style differs between the areas with better opportunities for outdoor activities, and the ones where these are poor. The results, derived by triangulation of methods, have confirmed the hypothesis assumptions. All three parts of the study have shown that the contribution of open space to the quality of life in highest in case of residents of Bonifacija. This is empirical evidence that the older, classically designed residential areas are superior to the new, contemporary ones. Open spaces in new residential areas are properly equipped only for the youngest children, while there are not enough sports fields and shaded areas. The worst situation was identified in Viška Sončava and Mesarska, where the observations revealed a large number of elderly residents outdoors, but their engagement was limited to accompanying children at play. 

The significance of determinants linked to open space is higher for residents belonging to youngest and eldest age, lowest income and education groups. There is an implication of these findings for urban planning. In a long term perspective of housing areas, their demographic and social structure inevitably changes. An example is provided by Nova Grbina: (four) playgrounds for youngest children are its prevailing outdoor facility, while after 14 years of its existence the youngest children represent only 12.00% of the residents. 

Linking our findings about good response of residents to spatial opportunities for active use of outdoor environment, and findings about importance of such life style from the literature [1,4,9,12,28], we can conclude that urban planning has the responsibility to respond. The fact that Bonifacija, the neighborhood from the “old times”, outperformed all the post transition areas in all three aspects of assessment is not a very encouraging message for the urban planners. Although the existing residents’ strong economic status, high level of completed education, and lower average age compensate for poor outdoor space and result in relatively positive perception of the quality of their living environment, the quality of life in new residential areas is inadequate for other users group and is not sustainable in the long term. The residential neighborhoods are not only built for active and affluent, who can execute their life style independently from their immediate surroundings, but have to provide healthy environment also for vulnerable groups, whose life style (in the case of children) or self-assessed health and well-being (in the case of elderly) depends also on physical and social activities performed in their close neighborhood [14,15,16,20]. All results of this study exceed the scope of this paper, therefore only key findings are presented. Multivariable analysis will be used for further analysis of results through various focal questions in the following stages of this study.

This research adds a health perspective to a considerable amount of convincing arguments in favour of introducing green areas in neighborhoods. The quality of open spaces is an important factor providing quality of life, in both regenerated areas as well as in new developments. The planning profession has one of the leading roles in ensuring this quality, since spatial plans do not only affect physical but also social and economic parameters [24,27]. Well-designed public open areas increase the value of surrounding properties to up to 5–7% and thus successfully attract new investors and the government for providing high quality urban design [42]. The densification of development to the cost of open space is therefore not economically justified, nor does it make sense from the perspective of public health, since it limits the opportunities for physically and socially active lifestyle.

## Supplementary Files

Supplementary File 1 Supplementary Table (XLS, 59 KB)
